# The Associations of Communal Space with Sense of Place and Mental Health in Public Housing: Evidence from Guangzhou and Hong Kong

**DOI:** 10.3390/ijerph192316178

**Published:** 2022-12-03

**Authors:** Tianyao Zhang, Jiahui Liu, Huiwei Chen, Mee Kam Ng

**Affiliations:** 1 School of Architecture, Harbin Institute of Technology (Shenzhen), Shenzhen 518055, China; 2 Faculty of Construction and Environment, The Hong Kong Polytechnic University, Hong Kong, China; 3 School of Architecture and Urban Planning, Guangdong University of Technology, Guangzhou 510090, China; 4 Department of Geography and Resource Management, Chinese University of Hong Kong, Hong Kong, China

**Keywords:** communal space, person–place process, sense of place, older adults, public housing

## Abstract

Communal space is regarded as essential for human well-being in high-rise developments in Asia and increasing attention has been given to the underlying mechanism of its effects in light of the ongoing COVID-19 pandemic. From the perspective of person–place processes, this paper explores ‘sense of place’ and its possible mediating effects on the relationship between communal space and the mental health of residents in high-rise public housing. An analysis of data from a questionnaire survey conducted in Hong Kong and Guangzhou revealed differentiated mechanisms according to local context and age group. Sense of place and its subcomponents mediated the connection between communal space and mental health in Hong Kong but not in Guangzhou. More specifically, place identity, place attachment and place dependence had stronger effects among older residents in HK than younger ones. The findings from this study can inform evidence-based planning and decision-making for public housing policy for health-oriented environments in high-density cities.

## 1. Introduction

The relationship between housing and health has come into the spotlight in interdisciplinary research, and housing has been acknowledged to be an important social determinant of health. There is growing interest in the health implications of public housing for its inhabitants, especially those with low incomes [1]. Public housing programmes worldwide aim to provide affordable shelter and to improve substandard living conditions; however, whether they can deliver health benefits to residents is debated [1]. Among all of the underlying health determinants of public housing, communal space plays a vital role because it is an extension of living space where residents can take part in physical activities and social interactions, both of which are clearly and significantly associated with individual health [2]. As a result of the lockdown measures taken during the COVID-19 pandemic, residential communal space has assumed an even more important role in urban environments. Providing a health-promoting environment to combat the potential health risks deriving from adapting to a new environment is a key challenge for public housing [3] and nurturing a sense of place by improving the quality of communal space may be an effective way to protect residents’ health.

The association of communal space and sense of place is particularly worthy of attention in the context of densely populated Asian cities such as Hong Kong (HK) and Chinese mega-cities. To house a growing urban population and meet the need to improve housing conditions, high-rise developments have dominated public housing in Chinese cities. However, due to the stigma attached to high-rise public housing and its association with concentrated poverty, it has been addressed as a pathogenic factor in Western urban scholarship [3,4]. The reported downsides of high-rise public housing in relation to health relate to the physical deprivation of contact with nature and the effects of social isolation from neighbours, such as loneliness, a reduced sense of belonging and even suspicion [4,5,6,7,8]. These effects may constitute partial explanations for the fact that public housing residents are more likely to report much poorer health conditions than the general population [1,9]. However, it is unclear whether the reported health risks of high-rise public housing exist in Hong Kong and other Chinese mega-cities, as residents in public housing in Hong Kong have reported high levels of satisfaction and Hong Kong’s housing developments have become world-renowned successful experiments that have been emulated by local governments in mainland China [3].

In the physical setting of high-rise public housing, communal space is particularly important for creating a liveable and healthy living environment [10]. The communal spaces of high-rise public housing can include outdoor and indoor public facilities such as green open space, meeting halls and so on. As both a physical setting and a place for social interaction, communal space provides a green public open space for residents to connect with nature and to take part in physical activities and social interactions. By enabling residents to establish social interaction, recognition and social ties, communal space can provide a nurturing ground for the development of a sense of place and sense of belonging, which can contribute to residents’ health and well-being [11,12,13]. Communal space can be perceived, evaluated, adapted and changed by residents in a whole person–place transactional process that may be causally linked with human health. Sense of place, representative of a person–place process, may serve as a vital bridge linking communal space and individual health. Therefore, examining the health implications of communal space through the intermediary role of sense of place is one way to understand the health effects of high-rise public housing in Chinese cities.

Using samples from Hong Kong and Guangzhou, this study explores how residents of public housing in China have developed a sense of place from communal space, and whether this person–place process is beneficial for residents’ mental health. Specifically, the study examines whether the intermediary role of sense of place in the relationship between communal space and mental health is different for different local contexts and population groups embedded in homogeneous cultural backgrounds. Therefore, it contributes evidence for whether and how communal space and sense of place decrease the health defects of high-rise public housing living environments that may impede mental health in China. To uncover the role of communal space and its mechanism of influencing mental health, the underlying person–place transactions are examined in terms of sense of place. By acknowledging the close ties between communal space and sense of place and their resultant implications on mental well-being, our findings could inform policy makers about the importance of providing health-oriented public housing environments for low-income and place-bound populations such as the older adults.

## 2. Literature Review and Research Hypotheses

Regarding the mechanisms underlying the effects of communal space on mental health, Suedfeld [14] noted the following:

*The environment has in fact no direct effect on human beings: rather it is filtered through their psychological and physiological information-processing systems.*

(p. 186)

Focusing on such information-processing systems, we believe that person–place interaction plays a vital role in building the relationship between communal space and mental health. Sense of place, which develops from an essential person–place process linking humans and their surrounding environment, makes a specific spatial setting memorable and pleasant for a person [15]. Human geographers including Relph [16] and Tuan [17] first conceptualised sense of place in the 1970s as deriving from human experiences that transform physical features into a place with meaning and identity. A place may become visually, socially, culturally and functionally unique to people when they have prolonged contact and experience with it [18,19].

Sense of place is a multivariate construct that depends on people’s interactions and activities in a physical setting [20,21] and which in turn shapes people’s subjective feelings and patterns of behaviour [22,23]. Accordingly, the three qualities that comprise sense of place—place identity (PI), place attachment (PA), and place dependence (PD) [24]—correspond to the three facets of the classical psychological construct of attitude [25], namely cognition, emotion and behaviour [26,27,28]. This analogy provides valuable insights into the person–place processes that nurture sense of place, from knowing and perceiving a place through investing oneself in a spatial setting, to growing an affective and emotional connection to it, and finally to loving the spatial setting so much that one would be willing to take actions to protect or defend it.

With regard to environmental factors determining a sense of place at the neighbourhood level, communal space (such as roads, entrances, parks, social and recreational facilities) has been proposed to be particularly important for nurturing a sense of place [29]. On the one hand, the physical attributes of parks, squares, roads and public facilities that are determinative for a nurturing ground for a sense of place [30,31,32,33]; on the other hand, the social processes within the neighbourhood are instrumental in the formation of attachment to and identification with a neighbourhood [34]. We thus propose that the physical attributes of communal place that invite people to socialise are sociopetal features, which are important for establishing a strong social network and a sense of belonging [35,36,37,38]. Additionally, residents who live adjacent to the open space or the amenities tend to know more neighbours and engage in more social activities, which are associated with increased attachment to a place [39]. It is thus important to combine physical elements, activities and meanings of the communal space to encourage people to sense and act for their own well-being and flourishing [40,41,42,43,44,45]. Empirical evidence from both Western countries and China have shown the positive effects of communal space on a sense of place and neighbourhood attachment [46,47]. Communal space can provide vital social interaction opportunities such as chance encounters between neighbours, fostering and strengthening community bonds [44]. In addition, the level of functional use of communal space can affect people’s emotional ties to their surroundings, particularly for place-bound populations such as the older adults who spend most of their time within their living settlements [48,49]. Some empirical evidence from Western context (the United States [50] and north Wales [51]) indicates that older people are more reliant on community amenities that are vital for improving their attachment to a place.

Sense of place can also help residents by building their social and emotional ties and improving their quality of life and overall health [45,52]. Within public housing programmes, sense of place is often considered a resource to revitalise public housing and to improve residents’ quality of life [24]. Numerous studies have demonstrated the positive impacts of sense of place on health by clarifying that positive emotional bonds to neighbourhood settings can provide psychological benefits that protect mental health, such as the development of self-identity, a sense of environmental mastery, residential satisfaction, a sense of belonging and happiness [13,53,54,55]. Sense of place can also shape health-related experiences, such as social support and social capital, which promote health by decreasing stress and improving coping strategies [10,56,57]. 

**Figure 1:**
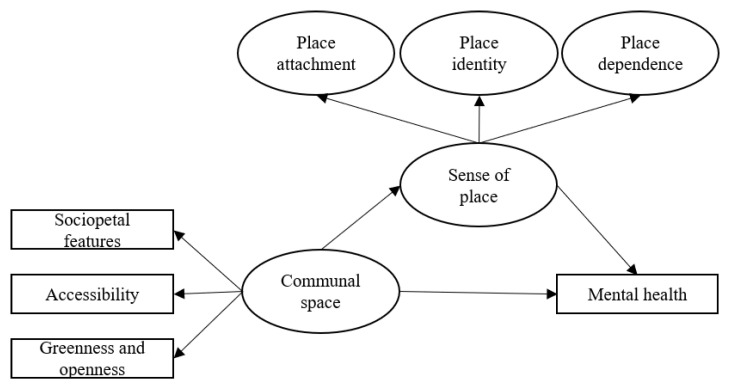
Conceptual framework of communal space, sense of place and mental health.

Accordingly, we posited that sense of place may play a mediating role in the health implications of communal space, which may explain the different health stories of public housing in different localities [10,58]. We therefore investigated whether the person–place process of sense of place could explain the relationship between communal space and the mental health of public housing residents, considering both locality and population differences and controlling for cultural difference (see Figure 1). Our hypotheses were as follows:

**Hypothesis** **1 (H1).**  *Communal space affects mental health via the intermediary factor of sense of place within the context of public housing*;

**Hypothesis** **2 (H2).**  *The mediating effects of sense of place differentiate according to the localities of public housing (HK and Guangzhou in our context)*;

**Hypothesis** **3 (H3).**  *The mediating effects of sense of place differ according to residents’ age*.

## 3. Methods

### 3.1. Data Collection

As person–place transactions are closely linked with localities where cultural embeddedness is of great importance [38,59,60], we chose HK and Guangzhou as study locations that share a homogeneous cultural background to control for the impacts of cultural variation. Although public housing programme in HK and Guangzhou has been implemented within different social, economic and institutional environments, HK’s experiences were introduced to Guangzhou in the 1990s when housing reform and marketisation were underway. It is reasonable to compare the pathways of the effects of communal space on sense of place and the resultant health implications in HK and Guangzhou, excluding the influence of cultural differences.

**Figure 2:**
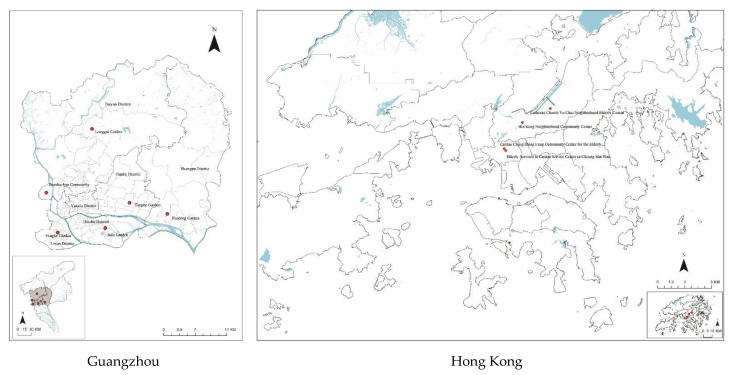
Location of the study area.

Focusing on conspicuous high-rise public housing, we selected housing cases by purposive sampling in the two cities, making sure their location and population size were comparable, as shown in Figure 2. For the HK survey, questionnaires were collected by arranging meetings with members of four community centres for older residents located near public housing estates in two districts—Sham Shui Po and Shatin, representing two typical urban forms of the older urban centres and new town in HK, respectively [61]. These community centres are places that older adults visit daily. We also interviewed adult residents in public parks near to these locations and completed the questionnaire with them on site, in order to involve both younger and older adults. In total, 459 valid questionnaires were collected in HK between July 2017 and December 2017. 

In Guangzhou, six typical public housing neighbourhoods were purposely selected across the urban central area and the inner suburbs, which are comparable to the locations of older urban centres and new town area in HK, respectively. We also regarded the development and construction time, population age structure, physical and design characteristics, building types and housing tenure types as the indicators of case selection. As a result, the selected neighbourhoods involve multi-storey buildings and high-rise buildings, public rental housing and subsidised owner-purchased housing, spanning different time periods from the late 1990s to the 2010s. Using the convenience sampling, structured face-to-face questionnaire surveys were conducted between May 2019 and June 2019 at communal spaces such as public squares, parks and community centres. The participants were adult residents who had been living in the housing for at least one year, and a total of 427 valid responses were collected. 

Photos were taken during field work to record the environment during both surveys. To explore the underpinning associations of sense of place with residents’ perceptions and evaluations of communal space, the interviewees were given the opportunity to provide further feedback that was not covered by the questionnaire. Semi-structured qualitative interviews were conducted synchronously on site, and 18 interview records that were highly relevant to our topic were transcribed.

### 3.2. Measures

#### 3.2.1. Mental Health

Mental health was measured using the Adult Mental Health Continuum Short Form (MHC-SF) (ages 18 or older) (Cronbach’s alpha = 0.916) [62]. The items described feelings and perceptions about self-cognition, interpersonal relationships, life and society, including ‘feel happy; feel interested in life; feel satisfied with life; feel that you had something important to contribute to society; feel that you belonged to a community; feel that our society is a good place, or is becoming a better place, for all people; feel that people are basically good; feel that the way our society works makes sense to you; feel that you liked most parts of your personality; feel good at managing the responsibilities of your daily life; feel that you had warm and trusting relationships with others; feel that you had experiences that challenged you to grow and become a better person; feel confident to think or express your own ideas and opinions; feel that your life has a sense of direction or meaning to it.

The respondents were asked to indicate how often they had the above feelings during the past month: ‘never’, ‘once or twice’, ‘about once a week’, ‘about two or three times a week’, ‘almost every day’ and ‘every day’. A mean score was computed to indicate overall mental health, with higher scores indicating better mental health. Latent class analysis was used to categorise the sample into five groups according to mental health scores (very good, good, neutral, poor, very poor).

#### 3.2.2. Sense of Place

Sense of place, comprising the three subcomponents of PI, PA and PD, was measured using 12 items on a 6-point scale (Cronbach’s alpha = 0.929). The respondents were asked to indicate how strongly they agreed with the 12 statements about person–place interactions (1 = strongly disagree, 6 = strongly agree). All 12 items were developed according to theoretical frameworks of sense of place and definitions of its three subcategories [27,63]. The construct validity of the scale was assessed using confirmatory factor analysis (CFA). The results indicated that the scale adequately identified the three aspects of sense of place (χ^2^/df = 5.613, *p* < 0.000; goodness of fit index (GFI) = 0.949; adjusted goodness of fit index (AGFI) = 0.920; root-mean-square error of approximation (RMSEA) = 0.072). Mean scores of all the 12 items and the items for the three subcategories were computed, with higher scores indicating stronger sense of place, PI, PA and PD, and all of these variables were treated as latent variables in structural equation models.

#### 3.2.3. Communal Space

We developed an 8-item, 6-point scale to measure the characteristics of communal space. The items described the residents’ perceptions of physical components constituting communal space, such as roads and streets, green space, playgrounds, parking lots and public transportation facilities. The respondents were asked to indicate how strongly they agreed with the statements on the quality of communal space, ranging from 1 = strongly disagree to 6 = strongly agree. Factor analysis was then performed and the eight items were aggregated into three principal components, namely (1) sociopetal features, (2) accessibility, and (3) greenness and openness. 

The validity of the communal space scale was assessed using CFA. The results suggested that construct validity was satisfied (χ^2^/df = 3.744, *p* < 0.00; GFI = 0.983; AGFI = 0.964; RMSEA = 0.056); the convergent validity was satisfactory, as the factor loadings were all greater than 0.6, the average variance extracted (AVE) of the latent variables ranged from 0.489 to 0.670 (the lowest acceptable value of AVE is 0.36 [64]), and composite reliability (CR) values ranged from 0.684 to 0.858, which is acceptable according to the benchmark of 0.6 [65]. The discriminant validity was also satisfactory, with all three latent variables significantly associated with each other, but the coefficient indices were less than 0.6 and less than the square root of AVE. The concept of communal space was then treated as a latent variable comprising three aspects in subsequent path analyses. 

#### 3.2.4. Control Variables

As sociodemographic variables may have important confounding effects on personal mental health, we included age, gender, marital status, educational attainment, occupation, household income, homeownership and length of residency as control variables in the path analyses.

### 3.3. Statistical Modelling 

Initially, descriptive statistics (mean, percentage, standard deviation) were calculated to understand the overall characteristics of the sample. Next, independent t-tests were performed to test whether there were significant differences between the respondents from HK and Guangzhou in terms of the quality of residential communal space, sense of place and mental health status. Next, to explore whether and how communal space could contribute to the development of sense of place and further improve residents’ mental health, we performed path analyses using SPSS AMOS (version 24.0) (IBM, Armonk, NY, USA). 

In addition, to compare pathways influencing the experience of living in public housing in HK and Guangzhou, we constructed two groups of structural equation models. As the age structures of the HK and Guangzhou samples were very divergent, we split the HK data into an elderly group and younger adult group using 65 years old as the cut-off and excluded the elderly group in Guangzhou’s dataset from further analysis because there were only five elderly respondents. Accordingly, we constructed two groups of structural equation models in two rounds: first, we examined whether sense of place played different intermediary roles between communal space and mental health among younger adults in Guangzhou and HK; second, we explored whether the associations of communal space with sense of place and mental health were different between the elderly and younger adults in HK. The two models both included one full model and three partial models. For all models, communal space and mental health were the independent variable and the dependent variable, respectively. The mediating variable in the full model was sense of place, and the mediating variables in the partial models were PI, PA and PD, respectively.

## 4. Results

### 4.1. Respondent Profile

The sociodemographic characteristics of the respondents (Table 1) show that there were great differences in the overall socioeconomic status of the HK and Guangzhou samples. Most of the respondents in Guangzhou were under 65 years old (98.8%), while around two-thirds of the respondents in HK were over 65 (61.4%). Consistent with the discrepant age distributions, the proportion of divorced/widowed respondents in HK (25.9%) was much higher than that in Guangzhou (2.1%). Approximately 80.7% of the respondents in Guangzhou had completed higher education, whereas in HK the proportion was as low as 34.6%, and about half (47.3%) of the HK respondents were poorly educated (junior secondary school or below). However, 72.9% of the younger participants in Hong Kong had a higher education qualification. The proportions of the respondents who were employed and unemployed were also reversed, with one-quarter of the respondents in Guangzhou being unemployed and a similar proportion being employed in HK (20.5%, 45.2% of younger respondents).

The distribution of monthly household income was more polarised in HK, with 39.2% of the HK respondents reporting less than HK$4000 and 46.4% reporting more than HKD 10,000, while in Guangzhou approximately half of the respondents (51.8%) had a monthly household income of HKD 4000–10,000. In contrast, 89.3% of the younger HK respondents had a monthly household income above HKD 10,000. The HK participants’ mean duration of residence was 19.96 years, which was much longer than that of the Guangzhou participants.

### 4.2. Comparison of Younger Residents in HK and Guangzhou

Guangzhou’s respondents gave significantly higher scores for the overall quality of communal space than their counterparts in HK did (MGZ = 4.22, SDGZ = 0.66; MHK = 4.02, SDHK = 0.77; *t* = 3.093, *p* = 0.002). The mean values of the three indicators of the quality of communal space ranged from 3.62 to 4.48 in Guangzhou and from 3.61 to 4.37 in HK. The score for sociopetal features was significantly higher for Guangzhou than for HK (*t* = 5.313, *p* = 0.000). For both sense of place and its three subcomponents, Guangzhou’s respondents scored significantly higher than their counterparts in HK, ranging from 4.37 to 4.50, indicating that younger residents in Guangzhou may have more positive cognition and emotional bonds towards and behavioural dependence on their neighbourhoods. Guangzhou’s younger respondents reported significantly better mental health than did those in HK (*t* = 9.873, *p* = 0.000; see Table 2).

**Figure 3:**
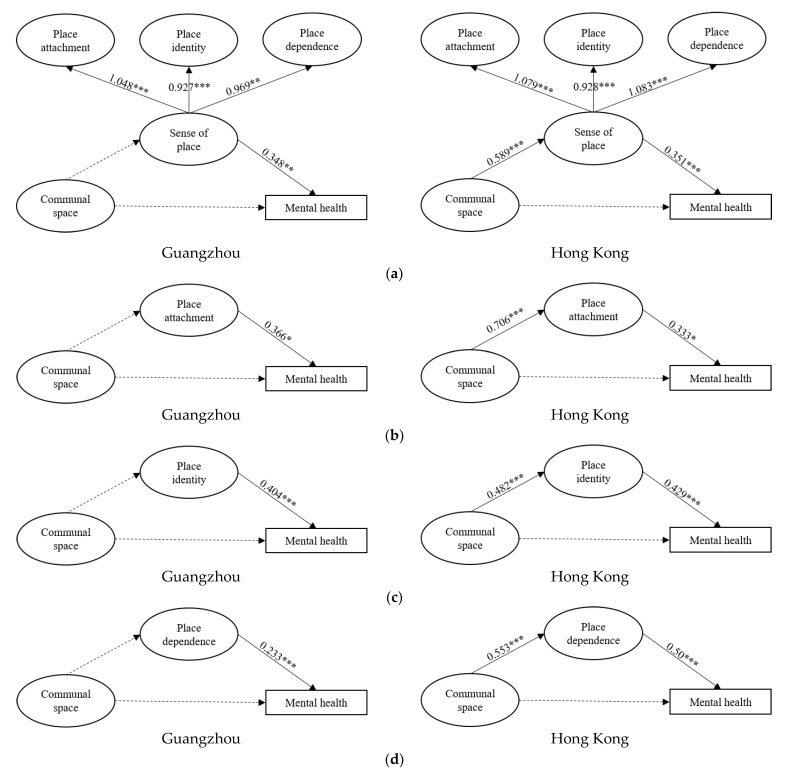
Pathways of model 1–4. (**a**) Model 1: Pathways of communal space linking sense of place and mental health in younger residents; (**b**) Model 2: Pathways of communal space linking place attachment and mental health in younger residents. (**c**) Model 3: Pathways of communal space linking place identity and mental health in younger residents; (**d**) Model 4: Pathways of communal space linking place dependence and mental health in younger residents. Note. *** *p* < 0.001, ** *p* < 0.01, * *p* < 0.05; the solid line is the significant path, but the dotted line is not.

To address the different intermediatory role of sense of place in the relation between communal space and mental health, four pairs of structural equation models were built with Guangzhou’s data and HK’s data. Prior to running the SEM, the assessment of normality was adopted. The Skewness and Kurtosis of univariates for each sample ranked from 0.060 to 1.493 and from 0.002 to 2.650, which were less than the critical values of 3 and 8, respectively, indicating that the data were normally distributed [66]. As the multivariate normality was not ideal, we used the bootstrapping method to reduce the statistical bias [67,68]. Most of the model fit indices (i.e., χ^2^/df, GFI, AGFI, TLI, CFI, and RMSEA) achieved the recommended threshold and thus reached an acceptable level of fitness (Table 3). Standardised coefficients and their statistical significance obtained from the path analysis are presented in Figure 3a–d, suggesting that neither sense of place nor its subcomponents played an intermediatory role between communal space and mental health in Guangzhou, although sense of place, PA, PI and PD did significantly contribute to mental health. A different pattern was identified in HK, where sense of place and its three subcomponents played a full intermediating role in the relation between communal space and mental health.

### 4.3. Comparison between the Older Residents and the Younger Residents in HK

As shown in Table 4, the quality of communal space and sense of place were rated significantly higher by older residents in HK than by younger ones. The mean scores for communal space given by the elderly ranged from 4.57 to 5.11, indicating a relatively good evaluation of the quality of communal space, while the mean scores of the younger residents ranged from 3.61 to 4.37. The average score for sense of place and its three subcomponents given by the elderly ranged from 4.67 to 4.77, suggesting a positive people–place interaction. The younger respondents had a much lower mean score for sense of place and its subcomponents (from 3.83 to 4.00). Additionally, the elderly reported significantly better mental health status (M = 4.65, SD = 0.82) than did the younger residents (M = 3.82, SD = 0.79). 

**Figure 4:**
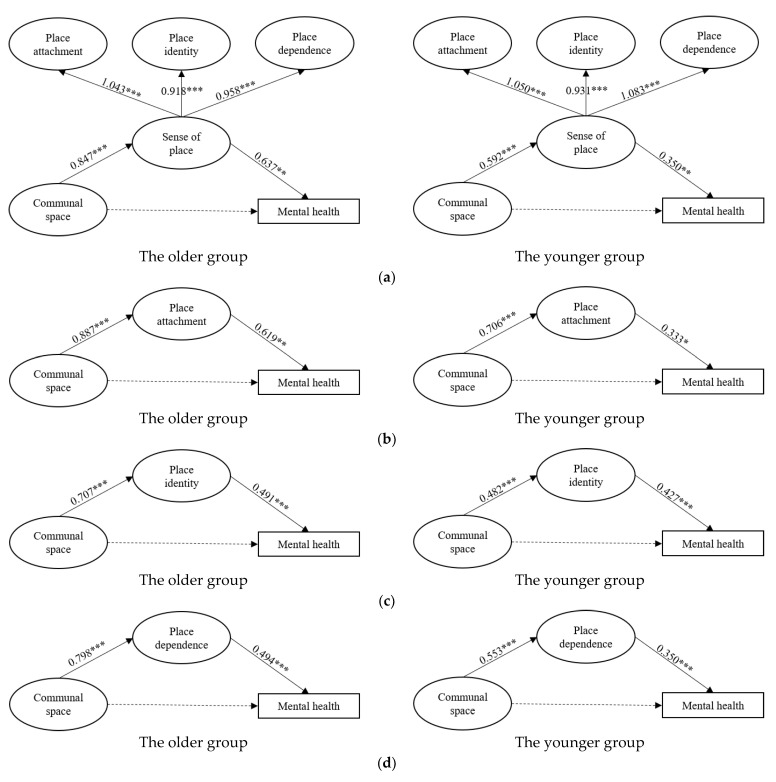
Pathways of model 5–8. (**a**) Model 5: Pathways of communal space linking sense of place and mental health in HK; (**b**) Model 6: Pathways of communal space linking place attachment and mental health in HK; (**c**) Model 7: Pathways of communal space linking place identity and mental health in HK; (**d**) Model 8: Pathways of communal space linking place dependence and mental health in HK. *** *p* < 0.001, ** *p* < 0.01, * *p* < 0.05; the solid line is the significant path, but the dotted line is not.

To examine whether the associations of sense of place with communal space and mental health differed between older and younger residents, four pairs of structural equation models were constructed using the HK data. All eight models achieved an acceptable level of fitness based on the model fit indices reported in Table 5. The results of path analyses are presented in Figure 4a–d. Similar patterns were found for the two age groups: both sense of place and its three subcomponents bridged communal space and mental health positively and significantly, while the coefficients of the paths for the elderly group were much higher than those for the younger group.

## 5. Discussion 

Our empirical findings indicate that sense of place significantly protects public housing residents from mental health problems in both HK and Guangzhou, for both older residents and their younger counterparts. In relation to Hypotheses 1 and 2, our results suggest that the health defects of high-rise public housing living are not apparent in HK’s case, and appropriately designed and maintained communal space does indeed nurture sense of place, which can effectively promote residents’ mental health. The results indicate that higher-quality communal space promotes the development of sense of place among HK public housing residents. 

The above pathway was more obvious for the older people than for younger residents, partially verifying Hypothesis 3. The older residents perceived higher quality communal space, had a stronger sense of place and were mentally healthier. One possible reason is that the older residents had lived longer in the community and the longer duration of residents resulted in a stronger sense of place, and people having a stronger sense of place display greater mental well-being. These pathways were observed in Ng’s study [61] on the two typical urban neighbourhoods in HK as well. Furthermore, enlightened by the extant literature in gerontology, we propose two underlying factors for better mental well-being: personal factors and life experiences, which have been ascertained to be influential in the overall quality of life of the older people [69]. In comparison to the younger people, the older people are more likely to have positive personal philosophies about life and a content and even-tempered disposition, which can contribute to a positive evaluation and interpretation of their surrounding environment. Through the experience of the first half of one’s life, the older people would develop an optimistic approach to life, such as being able to look forward and knowing the importance of acceptance and making the best of things [69]. Getting used to their living environment and developing a strong sense of place, the older people are more likely to develop a multi-faceted well-being.

However, the intermediary effect of sense of place in the relationship between communal space and mental health was not universal. In Guangzhou, no effect of sense of place was identified because communal space had no significant influence on sense of place. In comparison, sense of place (including PI, PA and PD) fully and robustly mediated the links between communal space and mental health in HK. Although the perceived quality of communal space in Guangzhou was reported to be better than that in HK, communal space failed to contribute to sense of place and residents’ mental health in Guangzhou. One possible reason is that the duration of residence of Guangzhou’s respondents was much shorter than that of their counterparts in HK (GZ–4.93 years compared to HK–19.96 years), and this leads to the fact that residents who have recently moved to the area may not feel a sense of place, echoing the previous argument that short living in the community makes it difficult to develop a sense of place [61,70].

To further understand the impacts of the perceived communal space on the sense of place, we examined the ways of people behaving in the space, suggesting a person–place transactional mechanism between communal space and sense of place [26,27,28]. Communal space serves as an important social place for daily life, which could combat potential health risks of high-rise public housing living, such as crowdedness and conflicts between family members [71]. This could be observed in HK’s data, as some interviewees stated as follows:

*My home is too small to stay in and I usually meet friends at the parks nearby. This makes me feel attached to the community.*

(Female respondent, 34, HK, 2019)

*I usually stay in the public space of the community, because I don’t want to face my daughter-in-law, who is always arguing with me about child-raising. So I usually go out on weekends when they she is at home to keep a stable mood.*

(Female respondent, 65, HK, 2019)

Thus, through the adaptively behavioural transactions with the communal space, the older residents in HK displayed a relatively high dependence on the communal space, which contributed to their sense of place accordingly. Comparatively, the younger respondents in Guangzhou told a different story about the person–place interactions within communal space. Guangzhou’s interviewees displayed relatively low aspirations for and passive behavioural transactions with the communal space, indicated by the following statements:

*I just jump rope in the communal space after work… but not often… since the space is always taken up by other residents, such as the older women who like dancing in the evening.*

(Female respondent, 37, Guangzhou, 2022)

*I usually take my child to play at the playground on the weekend… but it is very crowded, so I just watch him playing and don’t do anything else.*

(Female respondent, 32, Guangzhou, 2022)

*I sometimes walk along the community roads after dinner, but there is too much dog poo along the road… and the street light is too dim.*

(Male respondent, 43, Guangzhou, 2022)

These statements suggest that the communal space could not fully satisfy the use needs of the residents, in terms of both spatial design and the quality of the physical setting. The spatial inadaptability and low-quality setting reduce the usage of the space, decreasing the level of place dependence and attachment, which makes it difficult to develop a sense of place [72].

Regarding the quality of the communal space, our observations indicated that the communal space in Guangzhou’s public housing cases was not attractive enough to nurture residents’ emotional bonds with the neighbourhood or to construct self and group identities. Although the absolute score for perceived quality of communal space was higher for Guangzhou than for HK, the objective quality of communal space in Guangzhou did not necessarily seem to be better than that in HK’s public housing estates. Photos taken during our field trips suggest that the objective physical quality of the communal space in HK’s public housing was much better than that in Guangzhou (Table 6). Both the design and maintenance of the communal space in Guangzhou were not as good as in HK. Specifically, the design of physical elements in HK was more comfortable and appealing than in Guangzhou, such as the provision of sunshade and rain shelters, the vertical design of roof gardens and pedestrian lanes, and human-based considerations in recreational facilities. Thus, the divergence of subjective and objective evaluations of the quality of communal space between HK and Guangzhou may also suggest that residents evaluate the quality of communal space more highly when they are emotionally bonded with the space.

## 6. Conclusions

Our findings suggest that providing an effective physical ground for nurturing sense of place and embracing emotional bonding to a neighbourhood as well as a positive identity with it and behavioural dependence on it could be vital approaches to protect and promote the mental health of residents who live in public housing communities. This is especially important for public housing residents in Guangzhou due to the irrelevance of communal space to their sense of place.

Regarding person–place transactional processes, both physical design and post-occupancy evaluations of communal space should be re-examined by policy makers, focusing on their impacts on people’s cognition, affective emotion and patterns of behaviour, which are vital for developing a sense of place [26]. Although there are residential design guidelines in both mainland China and HK, such as the regulation of housing density and green ratios, human-oriented and health-promoting design interventions should be further enhanced. Additionally, personal and life experiences are influential during psychological information-processing, which embraces both interpretative and evaluative personal constructions of the environment [73]. Therefore, the needs and life experiences of residents in public housing programmes should be considered in terms of building a positive association between their communal space and sense of place.

Inevitable limitations of this study should be admitted: the cross-section design of this study limited its capacity to determine causality, so we could not provide certainty that the cause-and-effect relationships are correctly defined as to their direction. Additionally, the Guangzhou sample did not include older residents and it therefore may not fully reflect the overall picture of usage of communal space and its associations with sense of place. These issues should be addressed in a longitudinal study when we are able to collect data for several rounds. Moreover, self-selection effects may amplify the effects of work and family stress on mental health, which may restrict the potential effects of communal health on mental health. This should be considered when interpreting the mixed associations between communal space and mental health.

## Figures and Tables

**Table 1:** Statistics of the whole samples (%).

	Guangzhou (N = 427)	Hong Kong (N = 459)
**Age**		
Young (18–29)	32.8	12.9
Middle (30–64)	66.0	25.7
Old (≥65)	1.2	61.4
**Gender**		
Male	50.1	43.4
Female	49.9	56.6
**Marital status**		
Single	34.7	17.0
Married	63.2	57.1
Divorced/widowed	2.1	25.9
**Educational attainment**		
Junior secondary school and below	1.2	47.3
Senior secondary school	13.1	18.1
Junior college	40.0	15.9
University and above	45.7	18.7
**Occupation status**		
Employed	73.1	20.5
Unemployed	26.9	79.5
**Household income**		
<4000 HK$	18.0	39.2
4000–10,000 HK$	51.8	14.4
>10,000 HK$	30.2	46.4
**Home–ownership**		
Yes	20.4	29.2
No	79.6	70.8
**Duration of residence**	4.93	19.96

**Table 2:** Descriptive Statistics of the younger samples (N_GZ_ = 422, N_HK_ = 177).

Variables	Sample	M	SD	*t*-Test (df)	*p*
Quality of communal space	GZ	4.22	0.66	*t* (290) = 3.093	0.002 **
	HK	4.02	0.77		
Sociopetal features	GZ	4.36	0.88	*t* (597) = 5.313	0.000 ***
	HK	3.93	0.92		
Accessibility	GZ	4.48	1.00	*t* (378) = 1.411	0.159
	HK	4.37	0.87		
Greenness & Openness	GZ	3.62	1.21	*t* (597) = 0.096	0.924
	HK	3.61	1.05		
Sense of place	GZ	4.45	0.69	*t* (302) = 7.569	0.000 ***
	HK	3.94	0.76		
Place attachment	GZ	4.50	0.77	*t* (597) = 7.189	0.000 ***
	HK	4.00	0.79		
Place identity	GZ	4.37	0.74	*t* (597) = 7.943	0.000 ***
	HK	3.83	0.78		
Place dependence	GZ	4.48	0.79	*t* (281) = 5.791	0.000 ***
	HK	4.00	0.96		
Mental well-being	GZ	4.49	0.67	*t* (287) = 9.873	0.000 ***
	HK	3.82	0.79		

Note. N = number of participants, M = mean, SD = standard deviation; *** *p* < 0.001, ** *p* < 0.01.

**Table 3:** Model fit indices of model 1–4.

Model Fix		χ^2^/df	GFI	AGFI	TLI	CFI	RMSEA
Ideal threshold		<3	>0.9	>0.9	>0.9	>0.9	<0.05
Acceptable threshold		<5	>0.8	>0.8	>0.8	>0.8	<0.08
Model 1	GZ	2.608	0.899	0.861	0.850	0.881	0.062
	HK	1.625	0.862	0.807	0.901	0.923	0.060
Model 2	GZ	2.906	0.939	0.888	0.831	0.896	0.067
	HK	1.786	0.918	0.849	0.862	0.915	0.067
Model 3	GZ	2.372	0.951	0.911	0.866	0.917	0.057
	HK	1.417	0.934	0.878	0.923	0.953	0.049
Model 4	GZ	2.033	0.958	0.923	0.913	0.946	0.05
	HK	1.612	0.924	0.861	0.918	0.949	0.059

**Table 4:** Descriptive Statistics of HK samples (N_young_ = 177, N_old_ = 282).

Variables	Sample	M	SD	*t*-Test (df)	*p*
Quality of communal space	The older group	4.83	0.81	*t* (457) = 10.638	0.000 ***
	The younger group	4.02	0.77		
Sociopetal features	The older group	4.72	0.98	*t* (457) = 8.539	0.000 ***
	The younger group	3.93	0.92		
Accessibility	The older group	5.11	0.88	*t* (457) = 8.879	0.000 ***
	The younger group	4.37	0.87		
Greenness & Openness	The older group	4.57	1.11	*t* (457) = 9.158	0.000 ***
	The younger group	3.61	1.05		
Sense of place	The older group	4.84	0.81	*t* (457) = 11.800	0.000 ***
	The younger group	3.94	0.76		
Place attachment	The older group	4.86	0.80	*t* (457) = 11.377	0.000 ***
	The younger group	4.00	0.79		
Place identity	The older group	4.67	0.95	*t* (425) = 10.371	0.000 ***
	The younger group	3.83	0.78		
Place dependence	The older group	4.97	0.90	*t* (457) = 10.947	0.000 ***
	The younger group	4.00	0.96		
Mental well-being	The older group	4.65	0.82	*t* (457) = 10.617	0.000 ***
	The younger group	3.82	0.79		

Note. N = number of participants, M = mean, SD = standard deviation; *** *p* < 0.001.

**Table 5:** Model fit indices of model 5–8.

Model Fix		χ^2^/df	GFI	AGFI	TLI	CFI	RMSEA
Ideal threshold		<3	>0.9	>0.9	>0.9	>0.9	<0.05
Acceptable threshold		<5	>0.8	>0.8	>0.8	>0.8	<0.08
Model 5	The older group	2.811	0.859	0.808	0.834	0.867	0.080
	The younger group	1.714	0.857	0.802	0.883	0.908	0.064
Model 6	The older group	2.278	0.936	0.884	0.867	0.916	0.067
	The younger group	1.816	0.919	0.855	0.828	0.890	0.068
Model 7	The older group	2.130	0.942	0.896	0.863	0.913	0.063
	The younger group	1.450	0.934	0.881	0.899	0.935	0.051
Model 8	The older group	2.038	0.938	0.889	0.911	0.943	0.061
	The younger group	1.593	0.927	0.870	0.912	0.944	0.058

**Table 6:** Photo comparison of common open space between HK and GZ.

Element	HK Photo	GZ Photo	Comparison
Square	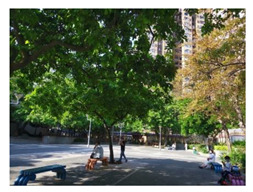	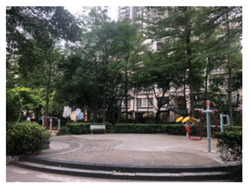	(1) More dedicated design of physical elements showed in HK’s cases (2) Better maintenance has been observed in HK’s cases (3) Better connectivity of common open space has been observed in HK’s cases.
Playground	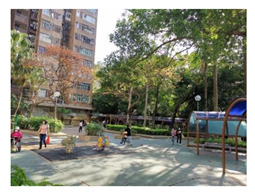	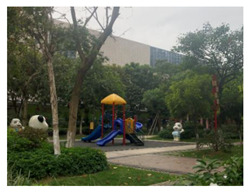	
Chatting space	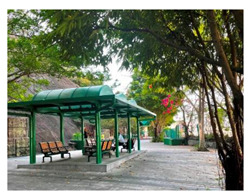	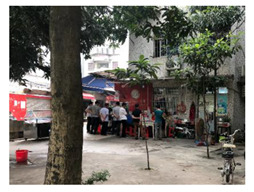	
Waking /jogging path	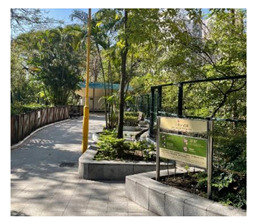	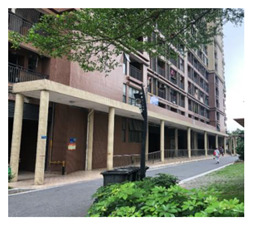	
Connectivity	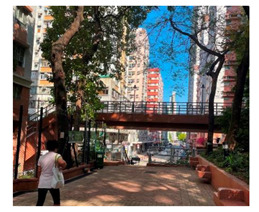	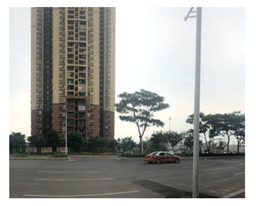	

## Data Availability

Data of the present study are available on request from the corresponding author.

## References

[1] Ruel E., Oakley D., Wilson G.E., Maddox R. (2010). Is Public Housing the Cause of Poor Health or a Safety Net for the Unhealthy Poor?. J. Urban Health-Bull. N. Y. Acad. Med..

[2] Dillman J.J., Dillman D.A. (1987). Private outside space as a factor in housing acceptability. Hous. Soceity.

[3] Yuen B., Yeh A., Appold S.J., Earl G., Ting J., Kwee L.K. (2006). High-rise living in Singapore public housing. Urban Stud..

[4] Freeman H.U.G.H. (1993). Mental Health and High-Rise Housing. Unhealthy Housing.

[5] Moser M.M., Herrenkohl R.C., Henn W., Norberg-Schulz C. (1981). Philosophy of Tall Buildings. Planning and Design of Tall Buildings.

[6] Costello L. (2005). From prisons to penthouses: The changing images of high-rise living in Melbourne. Hous. Stud..

[7] Fanning D.M. (1967). Families in flats. Br. Med. J..

[8] Cho S.H., Lee T.K. (2011). A study on building sustainable communities in high-rise and high-density apartments—Focused on living program. Build. Environ..

[9] Jargowsky P.A. (1997). Poverty and Place: Ghettos, Barrios, and the American City.

[10] Zhang Z., Zhang J.X. (2017). Perceived residential environment of neighborhood and subjective well-being among the elderly in China: A mediating role of sense of community. J. Environ. Psychol..

[11] Garling T., Golledge R.G. (1989). Environmental Perception and Cognition. Advance in Environment, Behavior, and Design.

[12] Chang J., Xie D., Huang J. (2015). Community Attachment Studies in Western Countries. Trop. Geogr..

[13] Fu Q. (2018). Communal space and depression: A structural-equation analysis of relational and psycho-spatial pathways. Health Place.

[14] Suedfeld P. (1991). Groups in Isolation and Confinement: Environments and Experiences. From Antarctica to Outer Space.

[15] Ghafourian M., Hesari E. (2018). Evaluating the Model of Causal Relations Between Sense of Place and Residential Satisfaction in Iranian Public Housing (The Case of Mehr Housing in Pardis, Tehran). Soc. Indic. Res..

[16] Relph E. (1976). Place and Placelessness.

[17] Tuan Y.-F. (1977). Space and Place: The Perspective of Experience.

[18] Campelo A., Aitken R., Thyne M., Gnoth J. (2014). Sense of Place: The Importance for Destination Branding. J. Travel Res..

[19] Chen R.B., Sekar A. (2018). Investigating the impact of Sense of Place on site visit frequency with non motorized travel modes. J. Transp. Geogr..

[20] Foote K., Azaryahu M., Kitchin R., Thrift N. (2009). Sense of Place. International Encyclopedia of Human Geography.

[21] Stedman R.C. (2003). Is It Really Just a Social Construction?: The Contribution of the Physical Environment to Sense of Place. Soc. Nat. Resour..

[22] Billig M. (2005). Sense of place in the neighborhood, in locations of urban revitalization. GeoJournal.

[23] Garnham H.L. (1985). Maintaining the Spirit of Place: A Process for the Preservation of Town Character.

[24] Tester G., Ruel E., Anderson A., Reitzes D.C., Oakley D. (2011). Sense of place among Atlanta public housing residents. J. Urban Health.

[25] Rosenberg M.J., Hovland C.I., McGuire W.J., Abelson R.P., Brehm J.W. (1960). Attitude Organization and Change: An Analysis of Consistency among Attitude Components.

[26] Fornara F., Bonaiuto M., Bonnes M. (2010). Cross-Validation of Abbreviated Perceived Residential Environment Quality (PREQ) and Neighborhood Attachment (NA) Indicators. Environ. Behav..

[27] Jorgensen B.S., Stedman R.C. (2001). Sense of place as an attitude: Lakeshore owners attitudes toward their properties. J. Environ. Psychol..

[28] Liu S.W., Cheung L.T.O. (2016). Sense of place and tourism business development. Tour. Geogr..

[29] Yu A. (2021). Open space and sense of community of older adults: A study in a residential area in Hong Kong. Int. J. Archit. Res..

[30] Cazzuffi C., Lopez-Moreno D. (2018). Psychosocial wellbeing and place characteristics in Mexico. Health Place.

[31] Farahani L.M., Lozanovska M.J.A.-I. (2014). A framework for exploring the sense of community and social life in residential environments. Archnet-IJAR.

[32] Karami S., Ghafary M., Fakhrayee A. (2014). Analyzing the correlation between urban spaces and place attachment Evidence from: Narmak neighborhood in Tehran. Eur. Online J. Nat. Soc. Sci. Proc..

[33] Shamsuddin S., Ujang N. (2008). Making places: The role of attachment in creating the sense of place for traditional streets in Malaysia. Habitat Int..

[34] Trentelman C.K. (2009). Place Attachment and Community Attachment: A Primer Grounded in the Lived Experience of a Community Sociologist. Soc. Nat. Resour..

[35] Hall E.T., Hall E.T. (1966). The Hidden Dimension.

[36] Lang J., Moleski W. (2016). Functionalism Revisited: Architectural Theory and Practice and the Behavioral Sciences.

[37] Dehkordi S.T., Soureshjani M.H. (2017). From sociopetal to sociofugal: A reverse procedure of Tehran urban spaces. J. Urban Des. Ment. Health.

[38] van Hees S., Horstman K., Jansen M., Ruwaard D. (2017). Photovoicing the neighbourhood: Understanding the situated meaning of intangible places for ageing-in-place. Health Place.

[39] Kim J., Kaplan R. (2004). Physical and psychological factors in sense of community—New urbanist Kentlands and nearby orchard village. Environ. Behav..

[40] McCunn L.J., Gifford R. (2014). Interrelations between sense of place, organizational commitment, and green neighborhoods. Cities.

[41] Inoue Y., Wann D.L., Lock D., Sato M., Moore C., Funk D.C. (2020). Enhancing older adults’ sense of belonging and subjective well-being through sport game attendance, team identification, and emotional support. J. Aging Health.

[42] Montgomery J. (1998). Making a city: Urbanity, vitality and urban design. J. Urban Des..

[43] Curley A.M. (2009). Draining or gaining? The social networks of public housing movers in Boston. J. Soc. Pers. Relatsh..

[44] Francis J., Giles-Corti B., Wood L., Knuiman M. (2012). Creating sense of community: The role of public space. J. Environ. Psychol..

[45] Wiles J.L., Allen R.E.S., Palmer A.J., Hayman K.J., Keeling S., Kerse N. (2009). Older people and their social spaces: A study of well-being and attachment to place in Aotearoa New Zealand. Soc. Sci. Med..

[46] Mason S.G. (2010). Can community design build trust? A comparative study of design factors in Boise, Idaho neighborhoods. Cities.

[47] Zhu Y.S. (2015). Toward community engagement: Can the built environment help? Grassroots participation and communal space in Chinese urban communities. Habitat Int..

[48] Ortiz A., Garcia-Ramon M.D., Prats M. (2004). Women’s use of public space and sense of place in the Raval (Barcelona). GeoJournal.

[49] Shabak M., Norouzi N., Abdullah A.M., Khan T.H. (2015). Children’s sense of attachment to the residential communal space. Procedia-Soc. Behav. Sci..

[50] Smith J.S., Cartlidge M.R. (2011). Place attachment among retirees in Greensburg, Kansas. Geogr. Rev..

[51] Burholt V., Naylor D. (2005). The relationship between rural community type and attachment to place for older people living in North Wales, UK. Eur. J. Ageing.

[52] Teo P., Huang S. (1996). A sense of place in public housing: A case study of Pasir Ris, Singapore. Habitat Int..

[53] Brown G., Raymond C. (2007). The relationship between place attachment and landscape values: Toward mapping place attachment. Appl. Geogr..

[54] Jorgensen B.S., Jamieson R.D., Martin J.F. (2010). Income, sense of community and subjective well-being: Combining economic and psychological variables. J. Econ. Psychol..

[55] Theodori G.L. (2001). Examining the effects of community satisfaction and attachment on individual well-being. Rural. Sociol..

[56] Bolam B., McLean C., Pennington A., Gillies P. (2006). Using new media to build social capital for health—A qualitative process evaluation study of participation in the CityNet project. J. Health Psychol..

[57] Prati G., Albanesi C., Pietrantoni L. (2016). The Reciprocal Relationship between Sense of Community and Social Well-Being: A Cross-Lagged Panel Analysis. Soc. Indic. Res..

[58] Zhang T.Y., Chiu R.L.H., Ho H.C. (2019). Suburban neighborhood environments and depression: A case study of Guangzhou, China. J. Transp. Health.

[59] Boese M., Phillips M.J.M. (2017). ‘Half of myself belongs to this town’: Conditional belongings of temporary migrants in regional Australia. Migr. Mobil. Displac..

[60] Glorius B., Kordel S., Weidinger T., Burer M., Schneider H., Spenger D. (2020). Is Social Contact With the Resident Population a Prerequisite of Well-Being and Place Attachment? The Case of Refugees in Rural Regions of Germany. Front. Sociol..

[61] Ng M.K., Yeung T.C., Kwan M.-P., Tieben H., Lau T.Y.T., Zhu J., Xu Y. (2021). Place qualities, sense of place and subjective well-being: A study of two typical urban neighbourhoods in Hong Kong. Cities Health.

[62] Keyes C.L.M. (2009). Atlanta: Brief Description of the Mental Health Continuum Short Form (MHC-SF). http://www.sociology.emory.edu/ckeyes/..

[63] Williams D.R., Vaske J.J. (2003). The measurement of place attachment: Validity and generalizability of a psychometric approach. For. Sci..

[64] Fornell C., Larcker D.F. (1981). Evaluating structural equation models with unobservable variables and measurement error. J. Mark. Res..

[65] Hair J.F., Black W.C., Babin B.J. (2009). Multivariate Data Analysis: A Global Perspective.

[66] Kline R.B. (1998). Structural Equation Modeling.

[67] Rong T. (2010). AMOS and Research Methods.

[68] Enders C.K. (2005). An SAS macro for implementing the modified Bollen-Stine bootstrap for missing data: Implementing the bootstrap using existing structural equation modeling software. Struct. Equ. Model.-A Multidiscip. J..

[69] Gabriel Z., Bowling A.N.N. (2004). Quality of life from the perspectives of older people. Ageing Soc..

[70] Williams A., Kitchen P. (2012). Sense of Place and Health in Hamilton, Ontario: A Case Study. Soc. Indic. Res..

[71] Gustafson P.E.R. (2001). Meanings of Place: Everyday Experience and Theoretical Conceptualizations. J. Environ. Psychol..

[72] Hashemnezhad H., Heidari A.A., Mohammad Hoseini P. (2013). Sense of place and place attachment. Int. J. Archit. Urban Dev..

[73] Stokols D., Shumaker S.A., Martinez J. (1983). Residential mobility and personal well-being. J. Environ. Psychol..

